# Impact of micronutrients supplementation on bone repair around implants: microCT and counter-torque analysis in rats

**DOI:** 10.1590/1678-775720150293

**Published:** 2016

**Authors:** Suzana Peres Pimentel, Renato Correa Casarin, Fernanda Vieira Ribeiro, Fabiano Ribeiro Cirano, Karla Rovaris, Francisco Haiter, Marcio Zaffalon Casati

**Affiliations:** 1 - Universidade Paulista, Faculdade de Odontologia, Pós-graduação em Odontologia, São Paulo, SP, Brasil.; 2 - Universidade Estadual de Campinas, Faculdade de Odontologia de Piracicaba, Departamento de Radiologia, Piracicaba, SP, Brasil.

**Keywords:** Micronutrients, Osseointegration, Tomography, Torque, Vitamin D

## Abstract

The use of natural substances and micronutritional approaches has been suggested as a therapeutic alternative to benefit the bone healing associated with no side effects. Nevertheless, the influence of micronutritional interventions with therapeutic proprieties on the bone repair has yet to be intensely evaluated, and no evidence is available exploring the impact of micronutrient supplementation on the peri-implant bone healing.

**Objective:**

This study investigated the effect of micronutrients supplementation on the bone repair around implants.

**Material and Methods:**

One screw-shaped titanium implant was inserted in each tibia of each rat, which were assigned to: daily administration, for 30 d, of the placebo solution (Placebo group-n:18) or micronutrients supplementation (Micronutrients group-n:18), based on calcium, magnesium, zinc, and vitamin D3 intake. After, the animals were sacrificed. One of the implants was removed by applying a counter-torque force to evaluate the force to rupture the bone-implant interface. The other implant was evaluated by microcomputed tomography (CT) examination to determine the bone-to-implant contact (BIC) and the bone volume (BV/TV).

**Results:**

No statistically significant differences were observed between the groups for both counter-torque values and microCT parameters (p>0.05).

**Conclusion:**

Within the limits of this study, micronutrients supplementation did not provide additional benefits to the bone healing around dental implants.

## INTRODUCTION

Bone metabolism homeostasis has been the motivation of numerous recent investigations. The events related to bone tissue repair are crucial to obtain a predictable bone restoration and also in optimizing osseointegration processes.

Implant therapy is an efficient type of dental rehabilitation that may fully and partially benefit edentulous patients by improving esthetic aspects, masticatory function, and dietary intake. Many implant modifications have been developed seeking to improve the long-term success rates of implants^12^. Additionally, different local and systemic substances have been studied to improve the bone healing in different clinical circumstances, although there are scarce evidences of safe and efficient agents to mediate the bone metabolism, especially in the osseointegration process.

Parathyroid hormone PTH(1-34) treatment increases bone healing, decreases bone resorption, and encourages bone repair during osteoporosis, providing a better peri-implant bone healing^29^, although it has been reported adverse events associated with its use such as nausea, cramps, headache, and hypercalcemia^20^. Similarly, while biphosphonates may avoid bone tissue resorption and improve bone deposition, their administration has been related to numerous adverse effects such as osteonecrosis of the jaws^16^. Consequently, the focus on therapeutic alternatives that could provide benefits to bone healing associated with no side effects, such as the use of natural substances and micronutritional approaches, has been explored.

In this context, our research group demonstrated that the regular use of resveratrol, a polyphenolic antioxidant present among others in the seeds of grapes and in the skin of black grapes, improved the repair of bone critical defects and the biomechanical retention of titanium implants^7^. In the last years, many other studies have revealed the potential therapeutic role of antioxidant and other micronutritional supplements (vitamins, minerals, trace elements) for bone metabolism, as in managing periodontal diseases^8^ ^,^ ^28^ or to prevent bone fractures^4^. For example, a recent review showed that the daily intake of some of these micronutrients could lead to additional benefits to treatment and prevention of periodontal disease in subjects presenting systemically deficient micronutrient^28^. Since periodontal disease could be associated with low serum/plasma micronutrient levels (which may result from dietary and/or life-style factors as well as nutrigenetic characteristics), supplementation has a promissory character in promoting benefits, although more studies are still necessary to reach a consensual protocol^28^.

Even though vitamin D and calcium are crucial for optimal skeletal development and maintaining bone mass, other macrominerals (e.g., magnesium) and trace minerals (e.g., zinc) are also important^26^ ^,^ ^32^. Studies support that magnesium supplementation provides increased bone mass in postmenopausal osteoporosis, during bone growth, and in other conditions^25^. Zinc also seems to be essential to bone turnover, presenting a stimulatory effect on osteoblastic bone formation and mineralization^32^. Meanwhile, it is important to highlight that the intake of dietary multivitamin/mineral supplements has been progressively increasing during the last years, with more than one-third of the overall population reporting the use of such supplements, in spite of diagnosis of nutrients deficiency^5^. However, to the best of our knowledge, no study assesses the impact of this supplementation in non-deficient conditions, especially regarding peri-implant bone healing.

Thus, in an attempt to improve the osseointegration process and considering the promising benefits that may be achieved by the micronutrients intake during the peri-implant bone healing, the aim of the current study was to determine the effects of micronutrients supplementation on bone repair around titanium implants. The hypothesis of this study was that the systemic daily intake of certain nutrients could positively benefit the bone healing and biomechanical retention of titanium dental implants.

## MATERIAL AND METHODS

### Animals

The animal cohort was composed of 36 10-week-old male Wistar rats. The rats were acclimatized for 15 d before use and kept in temperature-controlled cages (approximately 28°C), exposed to a 24-h light–dark cycle of equal time, in an air purification system, and had free access to water and food *ad libitum* (Labina, Purina^®^, Paulínia, SP, Brazil) in the Bioterium of Paulista University. According to the fabricant (Labina, Purina^®^), the composition of this regular diet includes approximately 13% of minerals – among them, each kg of diet contains 100 mg of calcium (calcium pantothenate), 4.400 UI of vitamin D3, and 110 mg of zinc. No magnesium is present in the formulation. The rats (*Rattus norvegicus*) included in the present study consume approximately 20 to 30 g of diet per day. The experimental procedure was approved by the Animal Care and Use Committee of the University (Permit Number: 139/12).

### Treatment groups

Animals were allocated in two groups: Placebo group (N=18), which received daily administration of a placebo solution for 30 d, and Micronutrients group (N=18), which received daily administration of 100 mg/kg of the animal of calcium (calcium citrate malate), 12 μg/kg of vitamin D, 6.72 mg/kg of magnesium (glycinate chelate), and 0.46 mg/kg of zinc (glycinate chelate)^1^ ^,^ ^17^ ^,^ ^22^. A stock solution of micronutrients was prepared and diluted in water for working concentrations. The placebo solution was composed of the same quantities of water as used in the preparation of micronutrients supplementation. The therapies were administered via gavage, with a 1 mL syringe, using 1 mL of the respective substances, for 30 d following surgery.

### Implant placement

**Figure 1:**
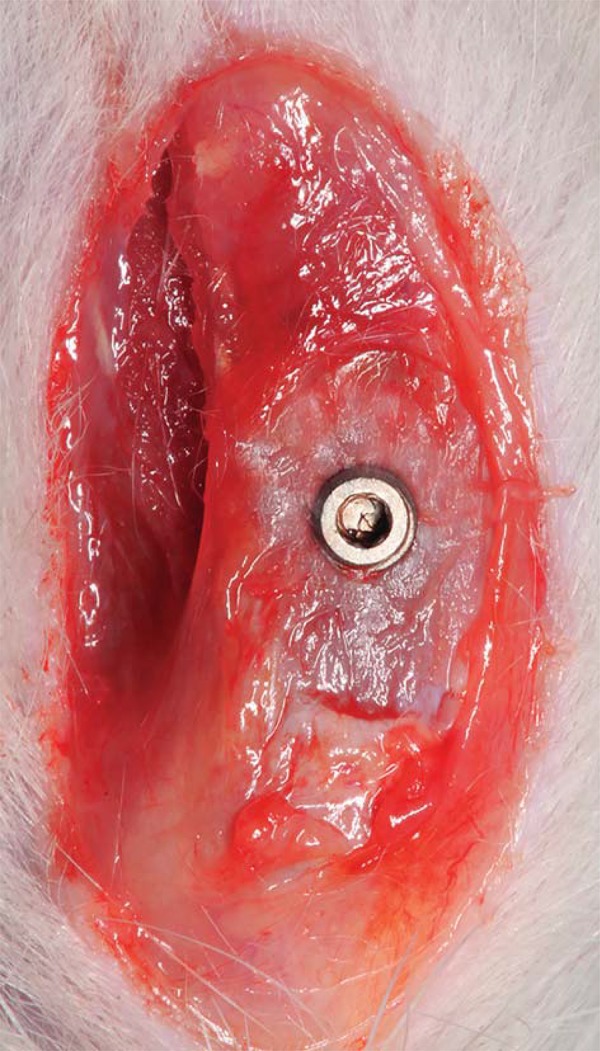
Illustration of the implant completely placed until all screw threads had been embedded into the bone cortex

General anesthesia was obtained by intramuscular administration of ketamine hydrochloride (0.5 mL/kg) and xylazine hydrochloride (10 mg/kg). During the surgical procedure, screw-shaped titanium implants with modified surface (blasted and acid-etched) (Implacil de Bortoli, São Paulo, SP, Brazil) were inserted in each tibia of each animal, according to a method previously described^7^. After the tricotomiae of tibiae and skin cleaning with iodine surgical soap, an incision of approximately 1.0 cm in length was made, and the bone surfaces of the tibiae were surgically exposed by blunt dissection. Under saline irrigation, bicortical implant beds were drilled at a rotary speed not exceeding 1500 rpm. A screw-shaped, commercially available pure titanium implant, 4.0 mm in length and 2.5 mm in diameter, was placed until the screw threads had been completely embedded in the bone cortex (Figure 1). Lastly, the soft tissues were replaced and sutured.

### Post-operative period

The animals were evaluated daily throughout the experiment to check for possible clinical or toxicological symptoms. Thirty days after the start of the study, the animals were euthanized by CO_2_ inhalation. One of the tibiae was dissected to expose the implant and torque force evaluation for implant removal. The other tibia (including the inserted implant) was removed and stocked in 70% alcohol for evaluation by microCT scans.

### Torque force evaluation for the removal of implants

One of the tibiae was dissected to expose the implant, allowing the attachment of a torque meter with a scale range of 0.1–10 N/cm and divisions of 0.05 N/cm (Mark-10, BGI, CA, USA). A wrench was adapted to the implant head to apply torque in the reverse direction of implant placement until complete rupture of the bone-implant interface was signaled by rotation of the implant. The torque force value obtained in N/cm was considered as the torque necessary for the breakdown of osseointegration^7^.

### Microcomputed tomography analysis

**Figure 2:**
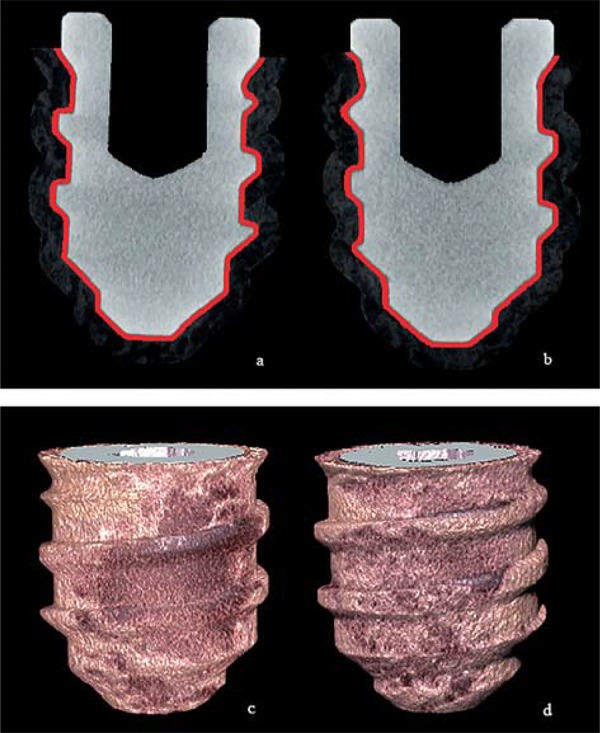
a and b) Representative coronal slice of Placebo (a) and Micronutrients groups (b), respectively. Red band represents the excluded layer around the implant (5 voxels). BIC parameter was calculated in addition to these 5 layers of voxels. Around the red band, note the assessed bone layer (20 voxels); c and d) Representative tridimensional images of assessed bone volume reconstructed using a computer program of Placebo (c) and Micronutrients groups (d)

Specimens obtained from the other tibia were evaluated by using microCT imaging. Samples were mounted on a custom attachment and scanned using a microCT scanner (SkyScan 1172; Bruker, Kontich, Belgium) at an isotropic resolution of 6.99 µm. Scanning was performed by 360° rotation with steps of 0.4 and 4 frames, using a 1 mm thick AlCu filter. The 900 projections were reconstructed using a tomographic reconstruction software (NRecon v.1.6.9.4, Bruker, Kontich, Belgium) with 2% of smoothing, 4% of ring artifact correction, and 10% of beam hardening correction, providing axial cross-sections. The projection image data were reconstructed to create 3D images, and they were analyzed using a computer program (CTAn v.1.12 software, Bruker, Kontich, Belgium). The following parameters were recorded for each volume of interest (VOI)^11^ ^,^ ^23^ ^,^ ^30^: (1) Bone-to-implant contact (BIC) – percentage of BIC along the threads of the implant surface; and (2) Bone volume fraction/density (BV/TV, i.e., Bone Volume/Total Volume) – percentage of the peri-implant bone volume to the total of the VOI. Because trabecular bone contains marrow cavities, the BV/TV should be <100%. The VOI corresponded to dilatation of the implant shape of 25 voxels: the inners 5 layers of voxels were discarded to avoid the artifacts observed in this region, resulting in a VOI of 7.933 mm^3^, and the top of the VOI was placed on the implant neck. The BIC was also calculated in outside of these 5 layers of voxels. Figure 2 illustrates the parameters evaluated. BV/TV and BIC were calculated on binarized images and all measurements were performed by the same calibrated masked examiner.

### Data analysis

Statistical analysis was performed using a software program (BioEstat 5.0, Sociedade Civil Mamirauá, CNPq, Tefé, AM, Brazil). Data were first examined for normality by the Kolmogorov–Smirnov test. For biomechanical evaluation of the retention of titanium implants and for microCT analysis, Student’s t test was used. The level of significance was set at 5%.

## RESULTS

The animals did not show any signs of systemic illness throughout the study period. The rats weighed 324±42 g at the beginning of the study and 402+31 g at the end in control group, and 332±41 g and 407+29 g at test group, respectively. No deaths were reported.

### Torque force evaluation

**Figure 3:**
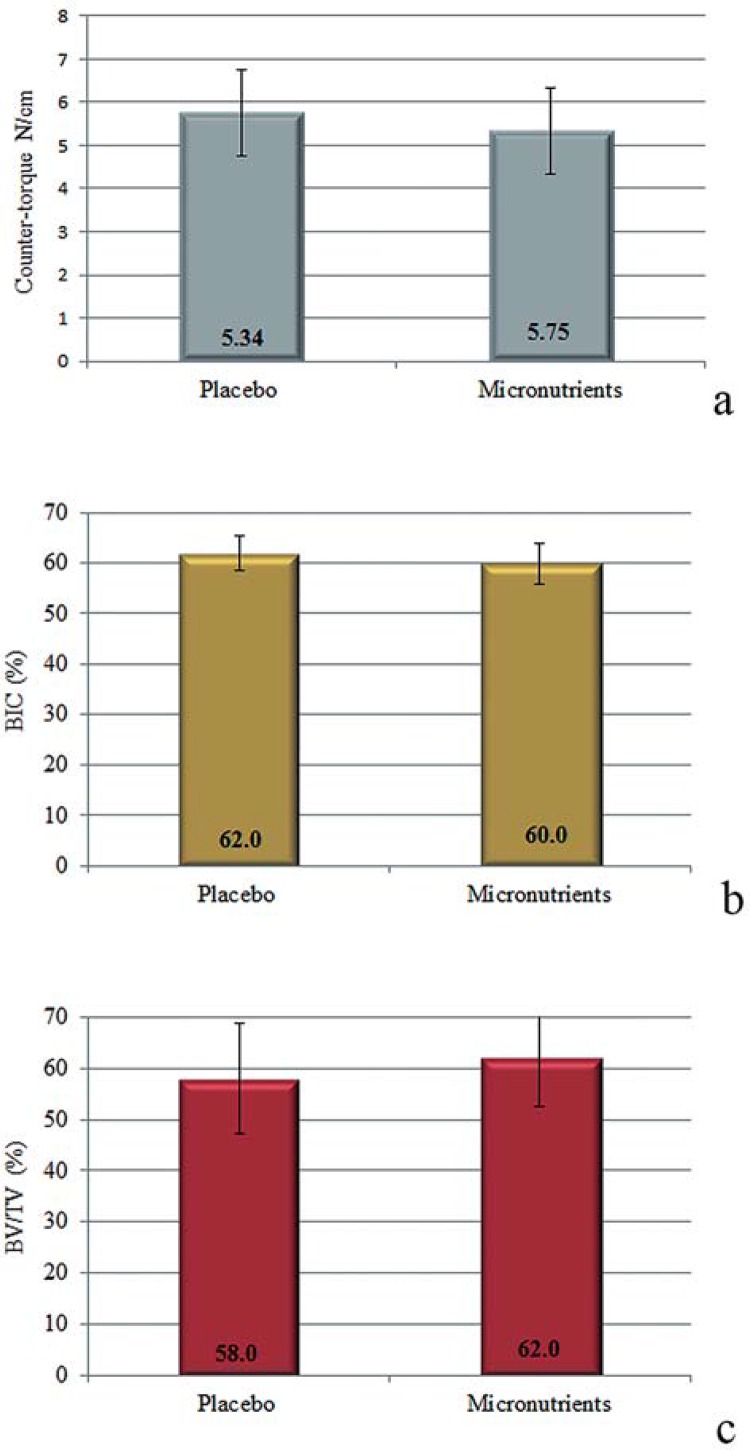
a) Graphic illustrating means and standard deviations of counter-torque values (N/cm) in both groups; b and c) Mean percentage and standard deviations of BIC (b) and BV/TV (c) in Placebo and Micronutrients groups. No significant differences between groups were observed for counter-torque and microCT analysis (p>0.05)

Data analysis demonstrated that micronutrients supplementation did not affect biomechanical retention of titanium implants. Intergroup comparison indicated no significantly differences between the counter-torque values for implant removal in Placebo group when compared to Micronutrients group (p=0.648). Figure 3a illustrates the values of counter-torque force in each group.

### MicroCT analysis

MicroCT evaluations indicate that the systemic intake of micronutrients supplementation did not affected the parameters evaluated, with no significant differences between groups for BV/TV (p=0.405) and BIC (p=0.232). Figures 3b and 3c demonstrate BIC and BV/TV values observed in each group, respectively.

## DISCUSSION

Vitamin D, calcium, and some macro and trace minerals are critical for maintaining bone metabolism^32^. Previous studies have demonstrated that, in bone tissues, vitamin D presents regulatory properties on osteocalcin and osteopontin, important markers involved in the maturation and bone mineralization stage, also modulating immune responses^26^. Vitamin D deficiency has been associated with bone loss, including periodontitis^2^, and has a negative effect on cortical peri-implant bone formation in ovariectomized rats, which may be compensated by vitamin D supplementation^33^. Indeed, human trials showed that vitamin D3 and calcium supplementation not only reduced hip bone resorption and fracture rates, but also improved vertebral bone density and total body calcium in post-menopausal women^9^. Magnesium also plays a key role in both bone tissue and mineral homeostasis, which may disturb the function of bone tissue cells, hydroxypatite formation, and interfere in mineral content. It was demonstrated that the deficiency of magnesium decreased the systemic bone density and the removal torque of osseointegrated implants^10^. Indeed, other studies have demonstrated that magnesium deficiency induces a decrease in bone formation, an increase in bone resorption, and impaired bone growth^25^. In addition, it has been reported that zinc is important to the maintenance of healthy bones, stimulating the reproduction and differentiation of osteoblastic cells and inhibiting osteoclastic activity in the bone tissue^14^. Bone growth retardation is also a common finding in various conditions associated with dietary zinc deficiency^32^.

Although there are some evidences concerning the role of these nutrients in bone healing in deficient animals/patients, including evaluations of bone repair around implants and periodontitis therapy, the effect of vitamin D and multiple minerals intake on the peri-implant healing in non-deficient conditions still has to be evaluated. Thus, this study investigated for the first time the effects of combined micronutrients supplementation on bone repair around titanium implants by using microCT and removal torque analysis. The results demonstrated that the systemic daily intake of micronutrients supplementation was not effective enough to positively benefit the early peri-implant bone formation and its biomechanical retention in non-deficient rats.

The findings of the present study are in accordance with Aral, et al.^3^ (2015), which also demonstrated that vitamin D3 and vitamin K2 supplementation were not effective in the reduction of alveolar bone resorption in rats with experimentally induced periodontitis. Conversely, Hong, et al.^13^ (2015) recently demonstrated in dogs that vitamin D3 and calcium supplementation during the early stage of socket healing promoted accelerate bone regeneration, improving new bone formation, bone density, and reducing the extent of vertical ridge resorption. Accordingly, Spolidorio, et al.^24^ (2010) showed that vitamin D and calcitonin reduced osteopenic changes in rats with alveolar bone loss induced by cyclosporine and the levels of tartrate-resistant acid phosphatase 5b (TRAP-5b) and pro-inflammatory cytokines – interleukin (IL)-1β, IL-6 and tumor necrosis factor (TNF)-α – induced by cyclosporine. Differences among studies may be attributed to the duration and dosage of supplements administration, variations in the experimental designs, and also to the nutrients combinations and parameters examined. In addition, the animals in this investigation received multiple nutrients supplementation only after the implant placement. Then, it would be relevant to consider if the prior administration of this supplementation – during a period before defect establishment or implant placement, besides the administration following surgery – could achieve more hopeful outcomes in terms of bone healing around implants.

Although the investigation of the impact of combined multi-nutritional component supplements during the peri-implant bone healing is important, the absence of evaluations of the effect of each nutrient individually could be considered a limitation of the current investigation. However, in the present investigation, the biological rationale to administrate calcium supplementation in addition to vitamin D was based in previous studies, including a recent meta-analyses^4^ that demonstrated that vitamin D alone is unlikely to prevent fractures, whereas supplements of vitamin D, in addition to calcium, are required for effectiveness, preventing increased fracture incidence. The additional supplementation with magnesium and zinc was also based on previous data, which supported that magnesium is also essential for the conversion of vitamin D into its active form and necessary for calcium absorption and metabolism, like zinc, whose deficiency prevents full absorption of calcium, limiting or preventing the bone health benefits of calcium plus vitamin D^21^ ^,^ ^27^.

Another aspect to be discussed regarding the results observed in the current study is that the animals were not systemically deficient regarding nutrients intake. Indeed, there are multiple mechanisms that may contribute to micronutrient deficiencies, and several studies have shown that supplementation in deficient animals could regulate its effects. It is well established that some clinical circumstances – certain pharmacological interventions, metabolic disease conditions, or systemic inflammations, as observed in patients with rheumatoid arthritis, osteoporosis, diabetes mellitus, or aging – may increase fracture healing time and the rate of complications, such as non-unions, impairing bone healing around titanium implant^34^. Thus, it could be suggested that, especially for patients in these risky situations, the use of micronutrients supplementation on a daily basis could have a favorable effect to increase success rates, improving the prognosis of titanium implants. In this context, vitamin D supplementation has reduced circulating concentrations of pro-inflammatory mediators in patients with immune system disorders or osteoporosis^15^ ^,^ ^19^. In accordance, Liu, et al.^18^ (2014) demonstrated recently that vitamin D supplementation was an effective strategy to improve the fixation of titanium implants in mice with chronic kidney disease, a common condition that leads to vitamin D deficiency. Nielsen, et al.^21^ (2011) also reported that zinc supplementation may be promising to bone health in postmenopausal women with deficient zinc intakes, but not in patients consuming adequate amounts of this mineral. However, corroborating our results, other data demonstrated that vitamin D intake alone or combined with calcium had no significant effect on serum cytokine concentrations of systemically healthy individuals^6^.

The intention in evaluating nutrients supplementation in non-deficient conditions is based on the fact that, nowadays, a large scale of the population daily intake these compounds, trying to obtain a healthier systemic condition and/or improve the results of their biological processes. A recent study showed that more than one-third of the general population declares to be an supplementation user^5^. Thus, our intention was prove if this supplementation – in normoreactive conditions – could improve osseointegration. However, based on our results and previous studies, it could be suggested that nutritional supplements may be beneficial when used by special populations that require additional dietary micronutrient intakes^31^. Thus, the cost-effectiveness profile of the multivitamin/mineral supplement use in the general population should be questioned, and clinical practitioners should be aware that while routine micronutrients supplements are unlikely to promote adverse effects, it remains unclear if this supplementation is advantageous for the general population.

In addition, the investigation of biological mechanisms to a possible up-regulation of key bone-related markers related to nutritional supplementation would be important to support (and, maybe, to change dosage and intake protocols) the therapeutic potential of micronutrients supplementation to stimulate bone healing around titanium implants.

## CONCLUSION

In conclusion, this study demonstrated that the administration of micronutrients supplementation in non-deficient conditions did not improve the BIC and bone volume around implants observed in the microCT analysis. These findings were also in accordance with the biomechanical retention of titanium implants.
